# Foreign Bodies in Ear and Nose

**Published:** 1879-03

**Authors:** 

**Affiliations:** Oxford, Ga.


### FOREIGN BODIES, IN EAR AND NOSE.

By A COUVERT M.D., Oxford, Ga.

Your present correspondent, when a beardless M.D. of twenty-one (21) years, and when some old folks said to him he looked young enough to be going to school instead of practicing medicine; in the vexation of his mind retorted, that (in our rural region) "a big beard would be worth more to him, than a bushel of brains." But many toilsome years have healed the defect and silenced the sarcasm. During that memorable period, he by accident, was present, when an old doctor laid a little negro on a table to extract a shot from her ear, politely requesting him to remain and aid in the operation. I, (to change the pronoun,) said modestly, perhaps doctor, if you will turn the shotted ear down and jar the head with the hand, the little heavy intruder will drop out. No! No! said the doctor, I can best extract it with a scoop, and he proceeded to work, the "nigger" to kicking and bawling, and the blood to flowing, and I to slipping out and away, not willing to share the honors of the "procedure," as neither of us had reputation enough for two. A young unprofessional man who remained in the room on other business, gave me the sequel. The doctor, after using scoops, some of them improvised for the occasion; tired and unsuccessful, turned over the girl's head, jarred it with his hand and out dropped the shot. As Dr. Paul F. Eve afterwards said, when informed of the facts, "the old doctor dropped out of the family and the young doctor dropped in." This case directed my mind to foreign bodies in the ear, and cases often occurred to test the mode that seemed most promising, namely, washing them out, which I long practiced, and I find of late is so highly approved by that distinguished gynecologist and surgeon, Dr. J. Marion Sims. He reasons with far more anatomical precision than I did. My process of reasoning was very simple. If I throw in a stream of water and the foreign body falls into the return current, it will come out, or at least within easy reach; the outflowing stream, though slower, is larger than the inflowing one, hence it will be more apt to strike it and bear it out. Experience verified this reasoning, whether a bug, or fly, or seed, or other light thing comes out, which it usually does, after a few injections. But if otherwise, the foreign body has probably adhered to, or becomes imbeded in the cerumen of the meatus, which may be washed out with tepid water. Sometimes, however, especially in old people, the indurated nucleus of cerumen, requires much time to soften and bring it out. Impaired hearing is sometimes thus caused, and thus cured.

The benefits of posture of head, are not to be disregarded in removing heavy bodies, nor of drawing back the ear so as to straighten the meatus, nor of so keeping the point of the syringe pressed to one side as not to obstruct the outflowing current with its freight of foreign matter; neither should it be inserted far into the meatus.

When I was a boy, I saw an old negro, ("Uncle Sawney,") blow a china-berry out of a little boy's nose, after a real doctor, (not one of your patent medicine breed,) had failed in spite of his expert manipulations and glittering instruments. It was thus done. The boy was held head and hands. Sawney's finger pressed the unobstructed nostril to prevent escape of air, his open mouth covering the whole of the boy's mouth, he "blew him up," and then with a sudden puff, brought out the berry, like a wad from a popgun. I came into the professisn remembering Sawney, and have removed in the same way, peas, corn, pretty-by-night seed and other things, without blood, bawling, pain or trouble; but as the operator gets a splash of nauseous secretions in his face, I magnanimously operate by proxy, usually; only supervising an operation, which presto confers the degree of doctor of *nose-ology* on several other Sawney's of different colors and races, who happen to be only lookers on "in Israel." I should add, this blowing by Sawney was done more than a half century ago, and I still follow it, *con amore.*

